# Supplementary material for: Mathematical models are a powerful method to understand and control the spread of Huanglongbing
Source: PeerJ. 2016 Nov 3;4:e2642. doi: 10.7717/peerj.2642 (PMC5101597; doi:10.7717/peerj.2642)
Supplement: Supplemental Information 1 [file peerj-04-2642-s001.pdf]

Mathematical models are a powerful method to understand and control the spread of Huanglongbing

Rachel A. Taylor, Erin Mordecai, Christopher A. Gilligan, Jason R. Rohr, Leah R. Johnson

August 26, 2016

Supplementary Article 1: Huanglongbing Model Details

Rachel A. Taylor, Department of Integrative Biology, University of South Florida
Erin Mordecai, Department of Biology, Stanford University
Christopher A. Gilligan, Department of Plant Sciences, University of Cambridge
Jason R. Rohr, Department of Integrative Biology, University of South Florida
Leah R. Johnson, Department of Integrative Biology, University of South Florida and Department of Statistics, Virginia Tech

Corresponding email: rataylor21@gmail.com

We provide full details of the model with the equations to outline how the pathogen is transmitted between trees and psyllids. We adapt a non-spatial model of vector-borne disease for malaria (Parham & Michael, 2010). In our model, Equations (S1.1)-(S1.9), citrus trees are categorized as either Susceptible, $S(t)$, Asymptomatic, $A(t)$, or Infected, $I(t)$, in which Infected implies the disease is detectable by symptoms; we assume Asymptomatic and Infected trees transmit the pathogen with the same probability. Adult psyllids are Susceptible, $S_V(t)$; Exposed, $E_V(t)$; or Infected, $I_V(t)$; where Exposed indicates that the psyllids are infected but are not yet able to pass the disease on to another tree. Once infected, psyllids remain so for their entire lifespan. Successful transmission between psyllid and tree can only occur when psyllids feed off the phloem of the tree; the feeding rate is independent of grove size thus transmission is frequency-dependent. We assume well-mixing between trees and psyllids. A very small rate of natural death of susceptible and asymptomatic trees occurs and we include roguing of infected trees; together these trees are categorized as Removed, $R(t)$. However, removed trees are immediately replaced in the grove by susceptible trees, keeping the grove size constant. Thus, the removed category exists to keep track of how many trees have been removed and replaced over time; it does not represent alive trees in the grove. We assume that the grove has 100% susceptible trees initially, with psyllids feeding freely from the trees. At time 0, we introduce one infected tree. We consider the change in numbers of susceptible, asymptomatic, infected and removed trees for the following 20 years to understand the effects of the initial infection on the whole grove.

$$\frac{dS}{dt} = -\frac{ab}{N} I_V S - rS + r(N - I) + r_1 I \tag{S1.1}$$

$$\frac{dA}{dt} = \frac{ab}{N} I_V(t-\tau) S(t-\tau) e^{-r\tau} - \gamma A - rA \tag{S1.2}$$

$$\frac{dI}{dt} = \gamma A - r_1 I \tag{S1.3}$$

$$\frac{dR}{dt} = r(N - I) + r_1 I \tag{S1.4}$$

$$\frac{dS_V}{dt} = \lambda F - \frac{ac}{N}(A + I) S_V - \mu S_V \tag{S1.5}$$

$$\frac{dE_{V1}}{dt} = \frac{ac}{N}(A + I) S_V - 3\phi E_{V1} - \mu E_{V1} \tag{S1.6}$$

$$\frac{dE_{V2}}{dt} = 3\phi E_{V1} - 3\phi E_{V2} - \mu E_{V2} \tag{S1.7}$$

$$\frac{dE_{V3}}{dt} = 3\phi E_{V2} - 3\phi E_{V3} - \mu E_{V3} \tag{S1.8}$$

$$\frac{dI_V}{dt} = 3\phi E_{V3} - \mu I_V. \tag{S1.9}$$

N is the total number of trees in the grove, which is kept constant at 1001. $V = S_V + E_{V1} + E_{V2} + E_{V3} + I_V$ is the total number of psyllids. We split the Exposed stage into three

compartments to more accurately represent the length of the exposure period. Following Lloyd (2001), using n compartments, in which the rate of leaving each compartment is $n\phi$, produces a Gamma distribution for overall psyllid progression to the infectious class with a mean rate of ϕ. The more compartments used leads to a Gamma distribution with lower variance around the mean. This is a useful alternative to fixed time delays, which can be problematic when parameters are temperature-dependent. We measure time in years so all rates are per year. a is the feeding rate of the psyllid on the trees, b is the probability that a susceptible tree becomes infected from contact with an infected psyllid, and c is the probability that a susceptible psyllid becomes infected from contact with an infected tree. Hence bc is the vector competence. We impose a time delay τ to represent the length of the exposed period when a tree is infected but not yet infectious. This time delay is long (approximately 6 months (Gottwald, 2010)), hence we use a fixed time delay of length τ to represent this more accurately than using a simple exponentially distributed exposure period. r is the natural death rate of susceptible and asymptomatic trees. Trees that are exposed may not survive the exposure period due to natural death, thus we include a discount term $e^{-r\tau}$ to correctly model how many trees move from susceptible to asymptomatic. Asymptomatic trees develop symptoms and move to the infected class with rate γ. The rate of removal of infectious trees by roguing is r_1. We assume all removed and dead trees are replanted with susceptible trees, hence the addition of these trees in equation (S1.1).

Adult psyllids have a fixed birth rate λ, which includes the development of eggs and nymphs. We include a term F to represent flush seasons. Development of psyllids is very closely connected to availability of flush, as eggs are laid on flush and nymphs remain on the same flush for their development period. Thus, the birth rate λ is defined as the number of adult psyllids produced on a single flush, and F determines how many flush are in the grove over the year. Flush is produced seasonally in sub-tropical regions, such as Florida, usually a major flush in spring and a minor flush in autumn. Following Chiyaka *et al.* (2012), we use a sinusoidal term to represent flush as two peaks each year. Based on Hall & Albrigo (2007), individual trees have 6 flush on average during the major flush season in spring but 3 flush during autumn. We set F to be

$$F = 3N(1 + \sin(4\pi t + \tfrac{\pi}{2})). \tag{S1.10}$$

This allows the number of flush patches to vary between 0 and 6 times the number of trees in the grove, twice each year with peaks in April and October (the year start is determined by the temperature function, see below).

The birth rate per flush patch, λ, can be expressed as the product of the number of eggs laid over a lifetime $\frac{EFD}{\mu}$ (where EFD is the number of eggs laid per female per year) and the probability the eggs survive to adulthood p_{ea}, over the average duration of the immature stages (Mordecai *et al.*, 2013). Hence,

$$\lambda = \frac{EFDp_{ea}MDR}{\mu} \tag{S1.11}$$

where MDR is the vector development rate, i.e. 1/time for a vector to develop from egg to adult. The death rate of psyllids is μ. ϕ is the development rate of the bacteria within the psyllid, determining the length of their exposure period.

We do not include invasions of psyllids from outside groves for simplicity. This is based upon the assumption that neighboring growers will coordinate spraying in their grove, in order to reduce the likelihood of invasion. Therefore, the size of the vector population can be modeled as a function of the demographic parameters. The probability of having V vectors at time t tends to a Poisson distribution with mean $\frac{\lambda F}{\mu}$ (Parham & Michael, 2010).

Mathematical models of disease systems often use R_0, the basic reproductive number, as a measure of disease prevalence. It is a measure of how many secondary hosts will become infected if one initial host is infected in a naïve population. The equation for R_0 for Equations (S1.1)-(S1.9) is given by:

$$R_0 = \left(\frac{Va^2bc}{N\mu} e^{-r\tau} \left(\frac{3\phi}{3\phi + \mu} \right)^3 \left(\frac{1}{\gamma + r} + \frac{\gamma}{(\gamma + r)r_1} \right) \right)^{1/2} \tag{S1.12}$$

$$= \left(\frac{EFDp_{EA}MDRa^2bcF}{N\mu^3} e^{-r\tau} \left(\frac{3\phi}{3\phi + \mu} \right)^3 \left(\frac{1}{\gamma + r} + \frac{\gamma}{(\gamma + r)r_1} \right) \right)^{1/2}. \tag{S1.13}$$

This equation for R_0 can be understood by considering how disease propagates through the system. It is dependent on the ratio of vectors to hosts (V/N), the number of successful contacts from tree to vector, due to the vector feeding on the tree (ac), how many infectious vectors survive the exposed period ($\left(\frac{3\phi}{3\phi+\mu}\right)^3$), and how many successful feeds those vectors make on trees (ab) over the lifespan of an infected vector ($1/\mu$), the proportion of those trees which survive the exposed period ($e^{-r\tau}$), and the average length of time infectious, which is given by the length of time asymptomatic ($1/(\gamma + r)$), plus the proportion of asymptomatic hosts that progress to be the infected class ($\gamma/(\gamma + r)$) multiplied by the length of time infected ($1/r_1$). One useful aspect of this definition is that if $R_0 > 1$, the epidemic will spread throughout the grove, whereas if $R_0 < 1$, the disease will die out. This is because on average, if $R_0 > 1$, one infected tree infects as least one other tree causing the disease to continue rather than die out.

Parameter Values

Our model aims to use realistic parameter values for each of the parameters, which necessitates analyzing how each of the parameters varies throughout the year due to environmental conditions. Psyllids are ectotherms and thus will be sensitive to fluctuations in temperature both daily and throughout the year. We include this in our model by incorporating the data presented in Liu & Tsai (2000), which determines life history parameters at different temperatures. Following the work of Mordecai *et al.* (2013), we expect thermal response curves to be unimodal, and left-skewed, since metabolic reaction rates tend to increase up to an optimal temperature, then decline due to protein degradation and other processes. However, as the data were limited, we allow for linear response curves. We fit thermal response curves for EFD, p_{ea}, MDR and μ assuming Brière, quadratic or linear functions, and determine the best fitting curve by the Akaike Information Criterion (AIC). For μ, we also used a data point from Hall *et al.* (2011) to determine the effect of freezing on longevity of psyllids.

The parameter fits are included in Table S1.1 with the data and best fit for the parameters plotted in Figure S1.1.

Parameter	Curve	Curve Parameters
p_{ea}	Linear	$a = 0.4719(0.103)$, $b = 0.0109\ (0.004)$
MDR	Brière	$c = 5.286 \times 10^{-5}(1.822 \times 10^{-5})$, $T_0 = 10.02(2.858)$, $T_m = 34.17(2.335)$
EFD	Brière	$c = 0.011(0.004)$, $T_0 = 13.049(3.231)$, $T_m = 30.845(0.896)$
$1/\mu$	Quadratic	$q = -0.14221(0.04717)$, $r = 4.31998(1.59958)$, $s = 31.25498(15.96673)$

Table S1.1: Thermal response fits for psyllid life history parameters. Brière $[cT(T - T_0)(T_m - T)^{1/2}]$, quadratic, $[qT^2 + rT + s]$ and linear $[a + bT]$ curves were fitted for each parameter, with Akaike Information Criterion (AIC) used to determine the best fit. Best fit values are listed with the standard deviations in parenthesis. See Table S1.2 for psyllid parameter definitions. We fit longevity ($1/\mu$) rather than the death rate (μ) for ease. Note that in this table the parameters are daily rates but for (S1.1)-(S1.9) we change to yearly rates (see Table S1.2).

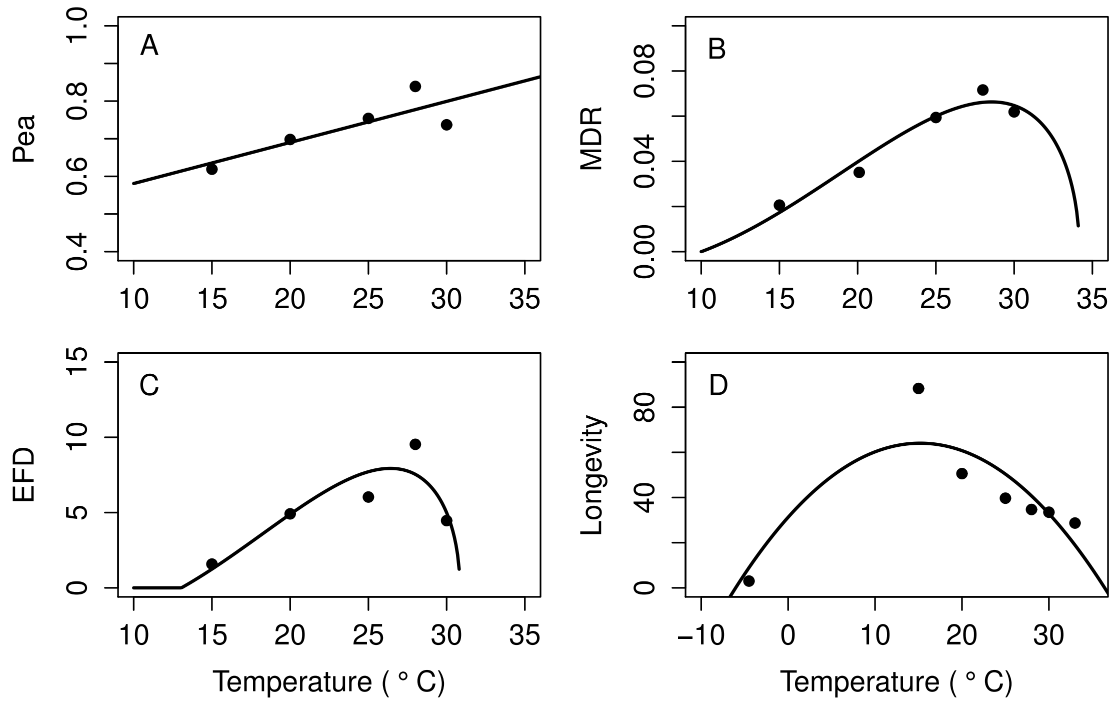

Figure S1.1: Thermal response curves for the psyllid life history parameters, using data from Liu & Tsai (2000). For D, we also used a data point from Hall *et al.* (2011) to determine the effect of freezing on longevity of psyllids. We fit linear, Brière, and quadratic curves for each parameter with the best fit (determined by AIC) shown here. In A, probability of egg to adult survival (p_{ea}); in B, psyllid development rate (MDR); in C, psyllid fecundity (EFD); in D, psyllid longevity ($1/\mu$). For parameter values of the fits, see Table S1.1. Note the different temperature range for D, due to the inclusion of a freezing reference point.

Note that we plot longevity (i.e., the inverse of the mortality rate; $1/\mu$) in Figure S1.1D. From Figure S1.1A, we see that the best fit for parameter p_{ea}, the probability of egg to adult survival, is a linear curve, which contradicts our current understanding of unimodal response curves as discussed earlier. Thus, more data collection on this parameter, for a wider range

of temperatures, would be useful to understand more clearly how it varies with temperature. However, we do not believe it is of much concern for our current model as Figure 3A (main text; sensitivity analysis) indicates that parameter p_{ea} is not influential on the model results.

A full list of all parameter values, with references, is provided in Table S1.2. All the parameters in this table are yearly rates, as used in equations (S1.1)-(S1.9).

We include temperature variation in the model over a year with the following equation:

$$T(t) = 25(1 + 0.3\sin(2\pi t)) \tag{S1.14}$$

which allows temperature to vary between 17.5°C and 32.5°C throughout the year, similar to average temperatures in Florida (Duever *et al.*, 1994). Our equation for Flush, (S1.10), determines seasonal flush in terms of time, but since temperature and flush are defined using sinusoidal functions, we can express flush in terms of temperature with the following equation:

$$F = 6N\left(1 - \left(\frac{10}{3}\left(\frac{T}{25} - 1\right)\right)^2\right). \tag{S1.15}$$

Including seasonal flush and temperature variation in the psyllid life-history traits results in R_0 changing throughout the year dependent on temperature (Figure S1.2). R_0 declines twice each year, both in summer and winter, but the higher temperatures are more deleterious and results in R_0 having a value of 0 for a significant portion of the year. At those high temperatures the psyllids are unable to produce any eggs. No transmission of infection would occur when $R_0 = 0$ and hence the number of infected trees will decline due to roguing during those times.

Sensitivity Analysis

We perform sensitivity analysis of R_0 for the temperature dependent parameters by analyzing the impact of these parameters on R_0. We calculate how each parameter contributes to the temperature sensitivity of R_0, $\frac{dR_0}{dT}$:

$$\begin{aligned}\frac{dR_0}{dT} &= \frac{\partial R_0}{\partial EFD}\frac{dEFD}{dT} + \frac{\partial R_0}{\partial p_{EA}}\frac{dp_{EA}}{dT} + \frac{\partial R_0}{\partial MDR}\frac{dMDR}{dT} + \frac{\partial R_0}{\partial \mu}\frac{d\mu}{dT} + \frac{\partial R_0}{\partial F}\frac{dF}{dT} \\ &= \left(\frac{1}{2EFD}\frac{dEFD}{dT} + \frac{1}{2p_{EA}}\frac{dp_{EA}}{dT} + \frac{1}{2MDR}\frac{dMDR}{dT} - \left(\frac{3}{2\mu} + \frac{1}{2\phi}\right)\frac{d\mu}{dT} + \frac{1}{2F}\frac{dF}{dT}\right)R_0\end{aligned} \tag{S1.16}$$

Here $\frac{\partial R_0}{\partial x}$ is the partial derivative of R_0 with respect to parameter x and $\frac{dx}{dT}$ is the derivative of parameter x with respect to temperature, using the equations in Table S1.2.

We use equation (S1.16) to analyze the relative contribution of each parameter to overall temperature sensitivity of R_0, as shown in Figure 3 in the main text. In the figure, we plot the full equation to indicate R_0's overall temperature sensitivity (black line) while each colored line is the contribution to temperature sensitivity arising from each parameter - e.g. the contribution of EFD is $\frac{\partial R_0}{\partial EFD}\frac{dEFD}{dT}$ whereas the contribution from μ is $\frac{\partial R_0}{\partial \mu}\frac{d\mu}{dT}$.

Parameter	Interpretation	Value	Reference
a	Feeding rate of vector	0.05*365	A
b	Probability of transmission from infected psyllid to susceptible tree	0.025	A
c	Probability of transmission from infected tree to susceptible psyllid	0.02	A
τ	Exposed time delay for tree	0.5	B
γ	Rate of developing symptoms	1/3	B
r	Death rate of susceptible tree	1/60	B
r_1	Removal rate of infected trees	1/7	C
F	Flush availability	$3N(1+\sin(4\pi t+\frac{\pi}{2}))$	D, E
L	Longevity (in days)	$-0.14221T^2+4.31998T+31.25498$	F, G
μ	Death rate of psyllids	$1/L*365$	-
ϕ	Development rate of bacteria within psyllids	36.5	A
EFD	Eggs per female psyllid per year	$0.0107*365*T(T-13)(30.8-T)^{1/2}$	F
p_{ea}	Probability of egg psyllid surviving to adulthood	$0.47192+0.0109T$	F
MDR	Development rate of psyllid	$(5.286\times10^{-5})*365*T(T-10.02)(34.17-T)^{1/2}$	F
ρ	Effectiveness of insecticide spray	0.6 - 0.99	H
m	Number of days spraying insecticide per year	10 - 60	J

Table S1.2: Parameter descriptions and values for the model described in Equations (S1.1)-(S1.9). T refers to temperature, as given by (S1.14). Rates are per year. References are: A - Pelz-Stelinski *et al.* (2010), B - Gottwald (2010), C - Martini *et al.* (2015), D - Chiyaka *et al.* (2012), E - Hall & Albrigo (2007), F - Liu & Tsai (2000), G - Hall *et al.* (2011), H - Rogers (2008), J - Spreen *et al.* (2006).

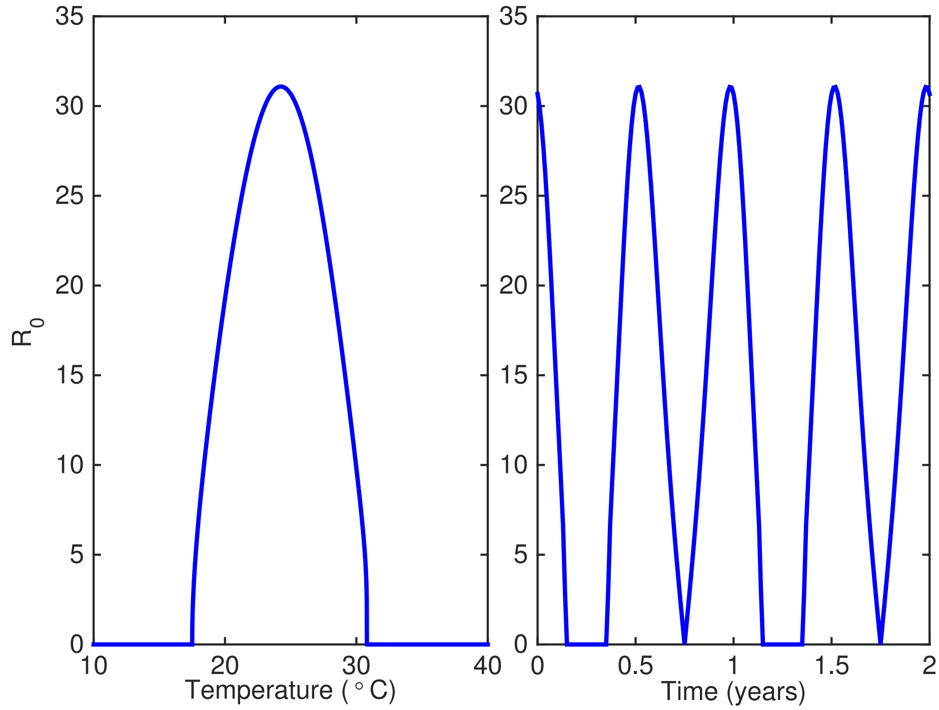

Figure S1.2: R_0 is plotted against temperature ($^{\circ}C$) and time (years) to show the seasonal fluctuations. The year begins in the middle of spring. R_0 is given by (S1.13) with parameter values as in Table S1.2 and temperature varying yearly according to (S1.14).

Insecticide Intervention

Insecticide spraying is currently the main method of control in widespread use in Florida (Qureshi *et al.*, 2014). The efficiencies of different insecticides vary, as well as the average duration of reduction of psyllids. The average insecticide efficiency is between 90-100% but some sprays can have an average efficiency as low as 53% (Qureshi *et al.*, 2014). When insecticide is applied to the groves it targets all adult psyllids through increasing their death rate. It will also reduce the birth rate of psyllids, to represent the insecticide killing eggs and nymphs. We analyse the effectiveness of the strategy using two aspects of the system: the peak number of psyllids infected and the total profit made. We include costs of the interventions to provide an aspect of cost-effectiveness as yields and profits are of primary interest to the citrus growers.

In our model, spraying occurs twice in a year, in spring and autumn. Both spring and autumn sprays will involve spraying for the same number of consecutive days. But between simulations we vary the total number of days spraying each year. In order to include insecticide spraying of m days in our model we use the following term:

$$ins(t) = \begin{cases} 365\rho & \text{if } t \in [t_n - \frac{m}{4*365}, t_n + \frac{m}{4*365}] \text{ or } t \in [t_n + \frac{1}{2} - \frac{m}{4*365}, t_n + \frac{1}{2} + \frac{m}{4*365}] \\ 0 & \text{else} \end{cases} \quad \text{(S1.17)}$$

where $ins(t)$ denotes the level of insecticide occurring at time t, t_n is an integer between 0

and 20 representing years within our simulation, ρ is the effectiveness of the spray (between 0 and 1) per day and m is the number of days in the year the insecticide is sprayed. Therefore, $\frac{m}{2}$ is the number of days in each spraying session. We multiply ρ by 365 to switch to a yearly rather than daily rate (to be consistent with (S1.1)-(S1.9)).

Insecticide spraying affects psyllids by killing them thus we include this term as an additional death rate. As we have a birth rate independent of the number of psyllids, we need to take into account that insecticide will reduce the number of psyllids and hence this rate should also decline. Thus, we multiply the birth rate of psyllids by a proportional term to represent this. This leads to the following equations for insecticide intervention:

$$\frac{dS}{dt} = -\frac{ab}{N} I_V S - rS + r(N - I) + r_1 I \tag{S1.18}$$

$$\frac{dA}{dt} = \frac{ab}{N} I_V(t - \tau) S(t - \tau) e^{-r\tau} - \gamma A - rA \tag{S1.19}$$

$$\frac{dI}{dt} = \gamma A - r_1 I \tag{S1.20}$$

$$\frac{dR}{dt} = r(N - I) + r_1 I \tag{S1.21}$$

$$\frac{dS_V}{dt} = \lambda \left(1 - \frac{ins(t)}{365}\right) F - \frac{ac}{N}(A + I) S_V - \mu S_V - ins(t) S_V \tag{S1.22}$$

$$\frac{dE_{V1}}{dt} = \frac{ac}{N}(A + I) S_V - 3\phi E_{V1} - \mu E_{V1} - ins(t) E_{V1} \tag{S1.23}$$

$$\frac{dE_{V2}}{dt} = 3\phi E_{V1} - 3\phi E_{V2} - \mu E_{V2} - ins(t) E_{V2} \tag{S1.24}$$

$$\frac{dE_{V3}}{dt} = 3\phi E_{V2} - 3\phi E_{V3} - \mu E_{V3} - ins(t) E_{V3} \tag{S1.25}$$

$$\frac{dI_V}{dt} = 3\phi E_{V3} - \mu I_V - ins(t) I_V. \tag{S1.26}$$

In the main text, we vary both ρ and m, the effectiveness of spraying and the total number of days spraying per year to understand the efficiency of insecticide at reducing disease prevalence.

Costs

The costs and profits of the insecticide intervention strategy is an important aspect in making informed decisions about the strategy to adopt. We focus on the key costs and profits in this early model to give a good indication of cost-effectiveness of the strategy (Table S1.3). The costs we include are: cost of removing a tree and planting a new tree (if the tree has died naturally or been removed due to infection) and cost of 1 day of insecticide spraying. We also include some basic profits, namely, the profits from susceptible, asymptomatic and infected trees over a year. The profits made from infected trees, in reality, will change as their

symptoms progress; we use an average value as we do not keep track of how long individual trees are infected for.

The cost of one day of insecticide spraying depends on the effectiveness of the insecticide, such that more effective insecticides cost more money to use. We use the following equation to determine cost:

$$\text{Insecticide cost per day} = \frac{15.075\rho}{1.203 - \rho}. \tag{S1.27}$$

This leads to a cost which varies from \$15 for $\rho = 0.6$ to \$70 for $\rho = 0.99$ where ρ is once again insecticide effectiveness.

One notable cost which we do not include is that of checking trees for infection. This can actually be rather costly depending on the number of days a year which are spent checking for infection and the method used for detection. However, there is such variation in how often people check trees, and the cost itself is difficult to calculate and will change between groves, that for this model we exclude it.

We include a discount factor by multiplying future costs and profits by the term $e^{-r_D t}$. This allows for more realistic results over a 20 year time period. Cost and profit values used in the model are included in Table S1.3. We have used profits and costs from Stansly *et al.* (2014) to assess likely values.

Parameter	Value
Discount factor r_D	0.001
Cost to replace tree	\$35
Cost of 1 day of insecticide spraying	Eqn. (S1.27)
Profit from susceptible/asymptomatic tree per year	\$388
Proportion of profit for infected tree	40%

Table S1.3: Cost and profit values used in our model. The discount factor is based upon a current inflation rate of 0.1%. The cost and profit values are estimated from Spreen *et al.* (2006); Stansly *et al.* (2014).

We incorporate the income calculations into our model by adding an additional differential equation:

$$\frac{dP}{dt} = 388(N - I) + (0.4)388I \tag{S1.28}$$

where $P(t)$ is the income at time t. We can then calculate costs and total profits using the solutions to the delay differential equations (S1.1)-(S1.9) and (S1.28), with the parameters in Table S1.3.

Methods

We numerically solve the delay differential equations (S1.1)-(S1.9) using Matlab, solver `dde23`. The grove has 1001 susceptible trees and 500 susceptible psyllids. At time 0, one of these 1001 susceptible trees becomes infected. That is, we are assuming that psyllids are already present but infection is not, before we start the simulation. This is in comparison to a case where psyllids invade an entirely susceptible grove, but some of the psyllids may already be infectious. Our scenario represents Florida well, since psyllids existed there for 7

years before HLB arrived, therefore it is likely that most groves had psyllids present before they became infected.

We analyze the dynamics of the infection over the following 20 years. For the intervention model, we apply the control strategy from time 0 and consider different control parameters while keeping the disease parameters the same as their values in Table S1.2. In no cases did applying the intervention strategy lead to complete removal of the disease from the grove.

References

Chiyaka, C., Singer, B.H., Halbert, S.E., Morris, J.G. & van Bruggen, A.H. (2012). Modeling huanglongbing transmission within a citrus tree. *Proceedings of the National Academy of Sciences*, 109, 12213–12218.

Duever, M., Meeder, J., Meeder, L. & McCollom, J. (1994). The climate of south Florida and its role in shaping the Everglades ecosystem. In: *Everglades: The ecosystem and its restoration* (eds. Davis, S.M. & Ogden, J.C.). St. Lucie Press Delray Beach, FL, USA, pp. 225–248.

Gottwald, T.R. (2010). Current epidemiological understanding of citrus Huanglongbing. *Annual review of phytopathology*, 48, 119–139.

Hall, D.G. & Albrigo, L. (2007). Estimating the relative abundance of flush shoots in citrus with implications on monitoring insects associated with flush. *HortScience*, 42, 364–368.

Hall, D.G., Wenninger, E.J. & Hentz, M.G. (2011). Temperature studies with the Asian citrus psyllid, Diaphorina citri: Cold hardiness and temperature thresholds for oviposition. *Journal of Insect Science*, 11, 83.

Liu, Y.H. & Tsai, J.H. (2000). Effects of temperature on biology and life table parameters of the Asian citrus psyllid, *Diaphorina citri* kuwayama (Homoptera: Psyllidae). *Annals of Applied Biology*, 137, 201–206.

Lloyd, A.L. (2001). Realistic distributions of infectious periods in epidemic models: changing patterns of persistence and dynamics. *Theoretical population biology*, 60, 59–71.

Martini, X., Pelz-Stelinski, K.S. & Stelinski, L.L. (2015). Absence of windbreaks and replanting citrus in solid sets increase density of Asian citrus psyllid populations. *Agriculture, Ecosystems & Environment*, 212, 168–174.

Mordecai, E.A., Paaijmans, K.P., Johnson, L.R., Balzer, C., Ben-Horin, T., Moor, E., McNally, A., Pawar, S., Ryan, S.J., Smith, T.C. & Lafferty, K.D. (2013). Optimal temperature for malaria transmission is dramatically lower than previously predicted. *Ecology letters*, 16, 22–30.

Parham, P.E. & Michael, E. (2010). Modeling the effects of weather and climate change on malaria transmission. *Environmental health perspectives*, 118, 620.

Pelz-Stelinski, K., Brlansky, R., Ebert, T. & Rogers, M. (2010). Transmission parameters for Candidatus *Liberibacter asiaticus* by Asian citrus psyllid (Hemiptera: Psyllidae). *Journal of Economic Entomology*, 103, 1531–1541.

Qureshi, J.A., Kostyk, B.C. & Stansly, P.A. (2014). Insecticidal suppression of Asian citrus psyllid Diaphorina citri (Hemiptera: Liviidae) vector of huanglongbing pathogens. *PloS one*, 9, e112331.

Rogers, M.E. (2008). General pest management considerations. *Citrus Industry*, 89, 12–17.

Spreen, T.H., Barber, R.E., Brown, M.G., Hodges, A.W., Malugen, J.C., Mulkey, W.D., Muraro, R.P., Norberg, R.P., Rahmani, M., Roka, F.M. & Rouse, R.E. (2006). An economic assessment of the future prospects for the Florida citrus industry. *Presentation to the Special Industry Task Force, Florida Department of Citrus, Lakeland, FL.*

Stansly, P.A., Arevalo, H.A., Qureshi, J.A., Jones, M.M., Hendricks, K., Roberts, P.D. & Roka, F.M. (2014). Vector control and foliar nutrition to maintain economic sustainability of bearing citrus in Florida groves affected by Huanglongbing. *Pest management science*, 70, 415–426.
