# Supplementary material for: Mathematical models are a powerful method to understand and control the spread of Huanglongbing
Source: PeerJ. 2016 Nov 3;4:e2642. doi: 10.7717/peerj.2642 (PMC5101597; doi:10.7717/peerj.2642)
Supplement: Supplemental Information 2 [file peerj-04-2642-s002.pdf]

Mathematical models are a powerful method to understand and control the spread of Huanglongbing

Rachel A. Taylor, Erin Mordecai, Christopher A. Gilligan,
Jason R. Rohr, Leah R. Johnson

October 6, 2016

Supplementary Article 2:
Additional Results for the Huanglongbing Model

Rachel A. Taylor, Department of Integrative Biology, University of South Florida
Erin Mordecai, Department of Biology, Stanford University
Christopher A. Gilligan, Department of Plant Sciences, University of Cambridge
Jason R. Rohr, Department of Integrative Biology, University of South Florida
Leah R. Johnson, Department of Integrative Biology, University of South Florida and
Department of Statistics, Virginia Tech

Corresponding email: rataylor21@gmail.com

In the main text we indicate how the number of trees and psyllids in their various categories changes over the first 8 years after infection arrives (Figure 2). We include here, Figure S2.1, the simulation for the full 20 year time span to show how it settles down to a steady state, with yearly seasonality. The figure for the full 20 years shows that by the end the total number of removed trees is over 1600, and the number of infected trees remains around 2/3's of the entire grove, while the remaining 1/3 of the grove is asymptomatic.

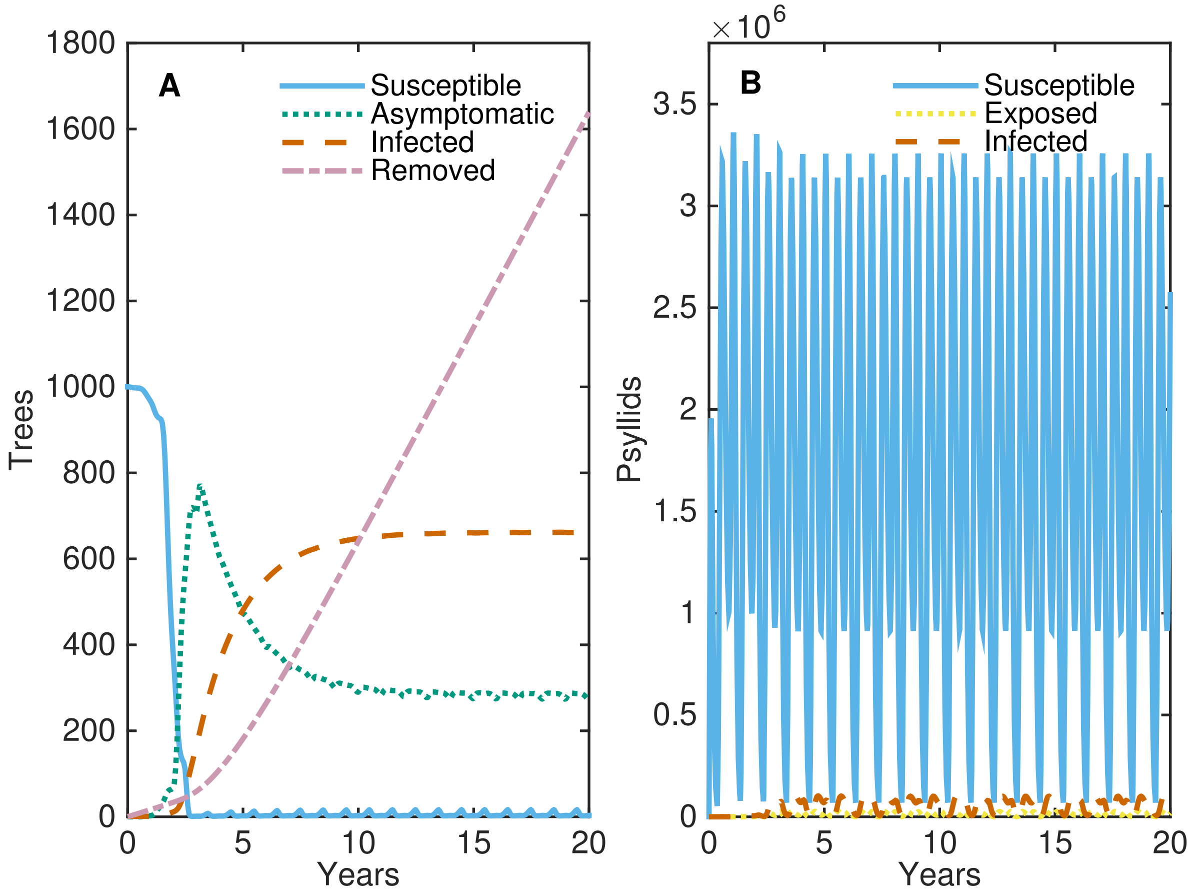

Figure S2.1: The changes in numbers of susceptible, asymptomatic and infected trees and susceptible, exposed and infected psyllids over 20 years when one tree is infected at time 0. In A, alive trees are either Susceptible (blue), Asymptomatic (green) or Infected (orange), and Removed trees are also plotted (purple). In B, psyllids are Susceptible (blue), Exposed (yellow) or Infected (orange).

In the main text, we also mentioned how it is roguing that prevents the infection from taking over the whole grove. If trees weren't rogued, all trees would remain infected rather than a cycle of replacing trees with susceptible trees which then become infected. We consider whether roguing at a higher rate, that is, reducing the average time until a tree is rogued, will affect the maximum number of infected trees and the total number of trees that need replaced (Figure S2.2).

Detecting infection and roguing trees sooner reduces the peak number of infected trees from about 800 to 350 trees with a faster decline when the average time until trees are rogued is shorter (Figure S2.2). This is a prodigious decrease in the number of infected

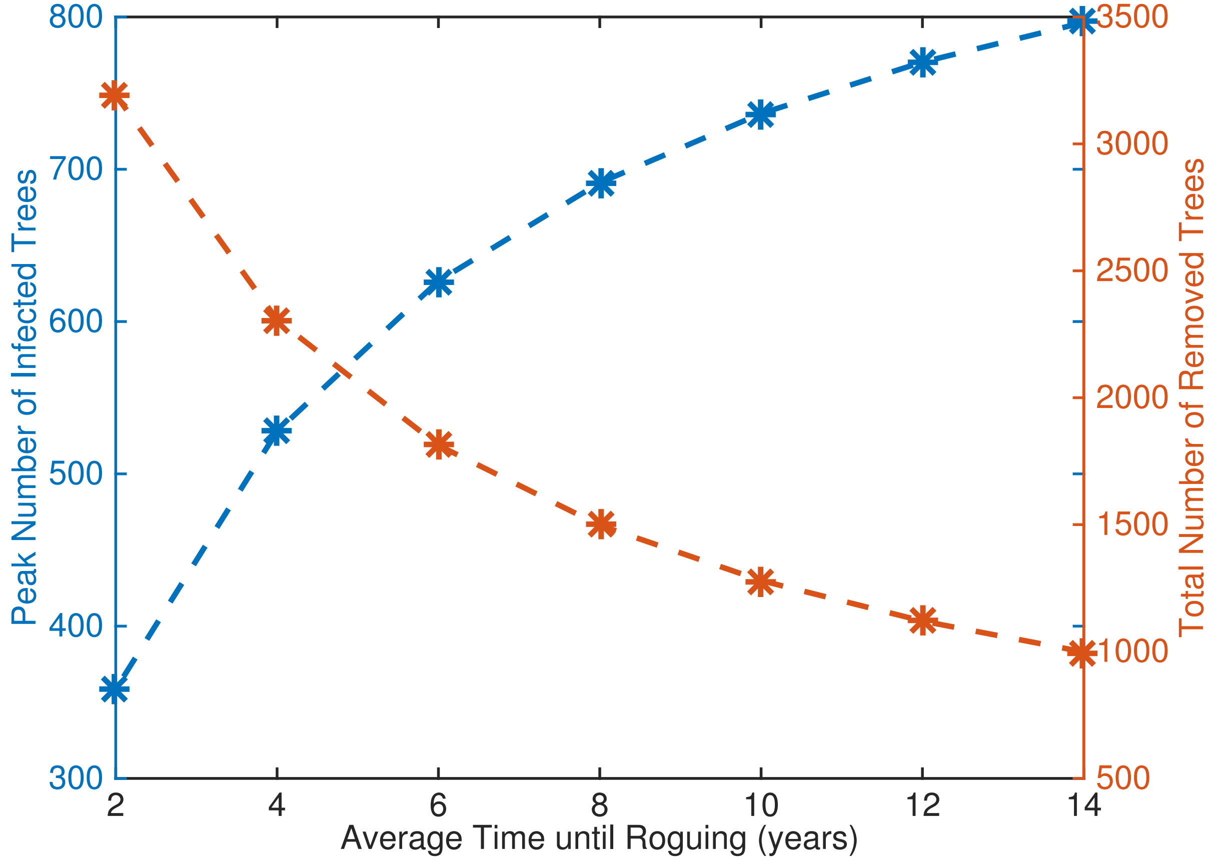

Figure S2.2: The effect of varying the average time from when an infected tree develops symptoms to when it is rogued on key features of disease spread. The mean time (in years) until an infected tree is rogued is varied from 2 to 14 years, thus the roguing rate (r_1) varies between 0.5 to 0.07 respectively. The peak number of infected trees (blue) and the total number of removed trees (orange) over a 20 year duration are plotted.

trees, especially when compared against the reductions provided by insecticide (see Figure S2.3). However, this is counterbalanced by an overwhelming increase in removed trees, from 1000 to 3300 trees, effectively replacing the whole grove more than 3 times within 20 years. These results are extreme but they serve as a reminder that the priorities of those intervening are of importance too. If the desired outcome was simply to eradicate the disease, the best strategy would be to rogue at the highest rate possible. However, in reality other factors are at play, such as the logistics of implementing the roguing strategy and the desire to maintain profits.

Sensitivity Analysis

We include here further details on the sensitivity analysis performed for the constant parameters. For each parameter p, we varied it by 10% (i.e. $0.9p$ and $1.1p$) and then calculated the value of R_0 at the temperature at which R_0 was maximum (Figure 3B, main text). We

output the low and high values for R_0 for each of these parameters to give more detail than the figure. The equation for R_0 is given in Article S1 as well as definitions of each of the parameters.

Parameter	Low R_0	High R_0
a	27.9780	34.1953
b	29.4914	32.6040
c	29.4914	32.6040
r_1	30.0813	32.2730
r	31.0000	31.1739
γ	30.7294	31.5173
τ	31.0737	31.0996
ϕ	30.7793	31.3420

Table S2.1: The low and high values of R_0 when each of the parameters p is changed to $0.9p$ and $1.1p$ as shown in Figure 3B. These are calculated when $T = 24.25$, when R_0 is at its maximum value of 31.09.

Insecticide Intervention

We include further details of our insecticide intervention results to aid in understanding the impact of insecticide application on the grove. In the main text, Figure 4, we plot both the peak number of infected psyllids and the total profit made over 20 years as both the number of days spraying and the effectiveness of the spray are varied. Here, we produce a similar plot but this time we use two different metrics of disease spread within the grove - the peak number of infected psyllids and the peak number of susceptible trees once infection has been present for a number of years and most trees are infected (Figure S2.3). The insecticide application allows the susceptible tree population to recover very slightly for a short time before the trees become infected again and the size of this recovery is what we measure by this metric.

In Figure 4 in the main text, insecticide application in spring and autumn results in significant reductions in the size of the infected psyllid population, especially when considered against the much higher population sizes when spraying is performed in summer and winter. When we focus on different metrics we can understand more clearly the effect on infection within the grove (Figure S2.3). Spraying in spring and autumn results in the peak number of infected trees lowering from 658 to 651 trees, which clearly is very small for a grove size of 1001. This is likely because the disease is so easily transmissible between tree and psyllid that only low numbers of infected psyllids are required to cause high levels of infection in the grove. However, when we consider the recovery of susceptible trees, we see that it increases from 26 to 42, a larger increase than the decline in infected trees. Therefore, reduced numbers of infected trees results in less asymptomatic trees also, and hence trees will remain susceptible for longer. This allows for more profits to be made on susceptible trees, and hence why small reductions in the number of infected trees produces much higher profits in the end. Once again, we can compare the results to the case of insecticide application in summer and winter, which are plotted on the same color scale. Figure S2.3C, D clearly highlights the lack of benefit of spraying at this time of year as no reductions in infected

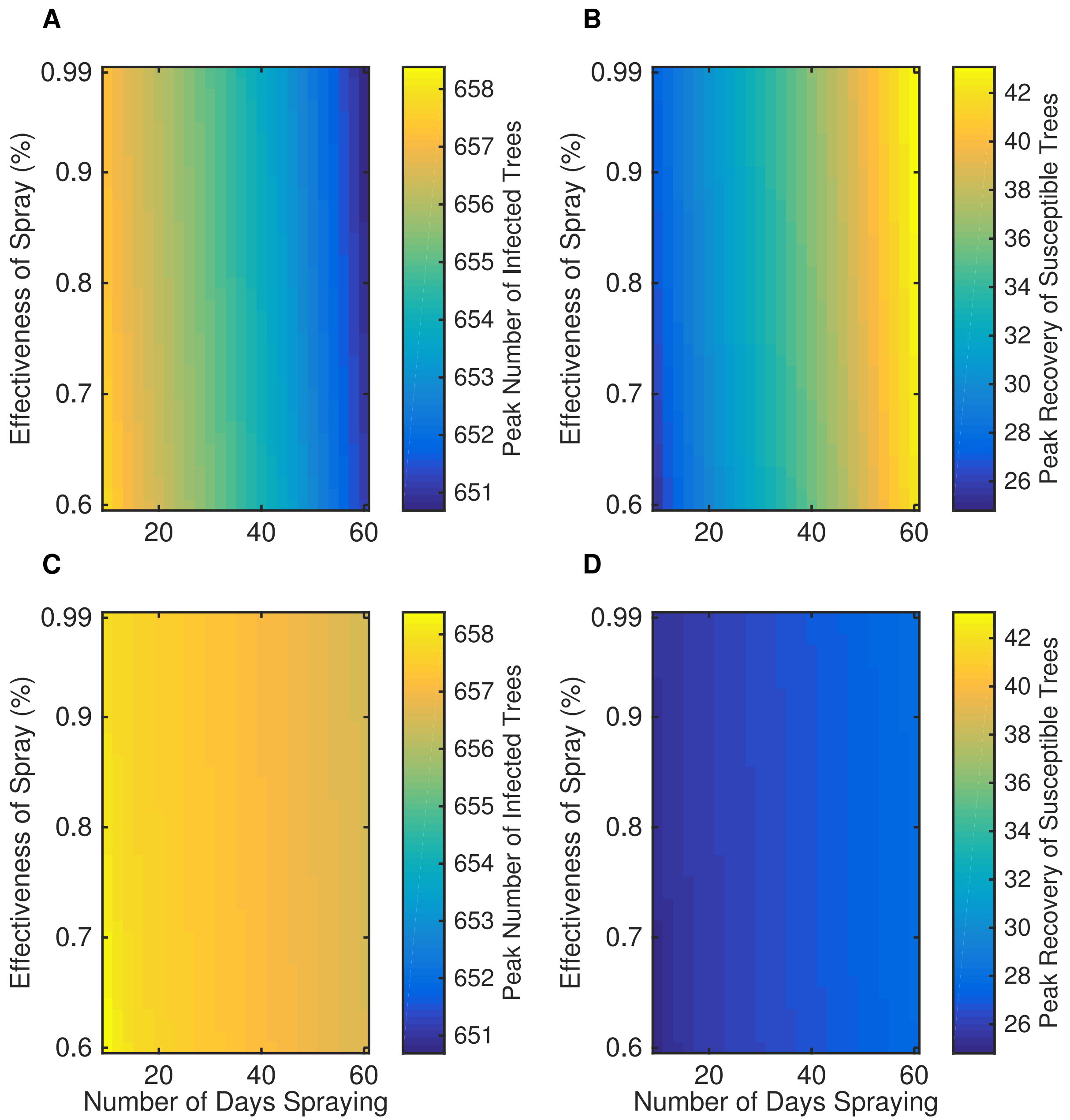

Figure S2.3: The effect of different insecticide strategies after a 20 year time span. Insecticide targets both the death rate and birth rate of psyllids. In A and B, the insecticide is sprayed in spring and autumn. In C and D, the insecticide is sprayed in summer and winter. In A and C, the peak number of infected trees is shown as a function of both the number of days spraying during each year and the effectiveness of the spray using the same color scale. In B and D, the peak number of susceptible trees during recovery periods is shown as a function of both the number of days spraying during each year and the effectiveness of the spray using the same color scale.The number of days spraying is the total per year, split equally between the two spraying regimes.

tree populations or increases in susceptible tree populations occur. This further supports our hypothesis that the seasonality of flush and temperature dependent traits of psyllids are

crucial when determining the impact and implementation of different intervention strategies.
